# Plasma Proteins and Platelets Modulate Neutrophil Clearance of Malaria-Related Hemozoin Crystals

**DOI:** 10.3390/cells9010093

**Published:** 2019-12-30

**Authors:** Sueli de Oliveira Silva Lautenschlager, Tehyung Kim, Danielle Lazarim Bidóia, Celso Vataru Nakamura, Hans-Joachim Anders, Stefanie Steiger

**Affiliations:** 1 Postgraduate Program in Pharmaceutical Sciences, State University of Maringá, Maringá, Paraná 5790, Brazil; lautenschlager@uem.br (S.d.O.S.L.); dlbidoia@gmail.com (D.L.B.); cvnakamura@uem.br (C.V.N.); 2 Division of Nephrology, Department of Medicine IV, Hospital of the LMU Munich, 80336 Munich, Germany; tehyung95@gmail.com (T.K.); hjanders@med.uni-muenchen.de (H.-J.A.)

**Keywords:** hemozoin, neutrophils, plasma, fibrinogen, platelet, malaria

## Abstract

Hemozoin is an insoluble crystalline pigment produced by the malaria parasite *Plasmodia* upon digesting host hemoglobin inside red blood cells. Red blood cell rupture releases hemozoin crystals into the circulation from where they are cleared by phagocytes such as neutrophils. We speculated that plasma proteins would affect the ability of neutrophils to clear hemozoin crystals. To test this, we cultured human blood neutrophils with hemozoin ex vivo and found that neutrophils ingested hemozoin (0.1–1 µm crystal size) in a dose-dependent manner into phagosomes and vesicles/vacuoles, resulting in morphological changes including nuclear enlargement, and vesicle formation, but not cell membrane rupture or release of neutrophil extracellular traps. The presence of human plasma significantly inhibited the ability of neutrophils to ingest hemozoin crystals. Platelet-poor plasma further inhibited the uptake of hemozoin by neutrophils. Selective exposure to fibrinogen completely replicated the plasma effect. Taken together, neutrophils cleared hemozoin crystals from the extracellular space via endocytosis into phagosomes and vesicles without inducing the release of neutrophil extracellular traps. However, human plasma components such as fibrinogen limited hemozoin clearance, whereas the presence of platelets augmented this process. These factors may influence the pro-inflammatory potential of hemozoin crystals in malaria.

## 1. Introduction

Malaria is a life-threatening disease caused by the *Plasmodium* parasite [1]. Globally, approximately 216 million cases of malaria were reported in 2016 with an estimate of 445,000 deaths [1]. Upon parasite (called sporozoite) transmission to humans through the bites of infected mosquitoes, the parasites distribute via the bloodstream to the liver. Within the liver, each sporozoite multiplies into thousands of merozoites that reach back into the bloodstream, where they infect red blood cells for further replication. Parasite replication occurs in a cyclic fashion within red blood cells. During this erythrocytic cycle, the parasite degrades hemoglobin into amino acids and heme inside the digestive vacuole, where the heme monomer is further oxidized into a toxic inert biocrystalline form called malarial pigment or hemozoin (HZ) [2,3,4]. Upon red blood cell rupture, HZ as well as other parasite toxins including *Plasmodium* DNA and glycosylphosphatidylinositol are released into circulation and recognized by pattern recognition receptors expressed on phagocytes and other immune cells in the blood and tissues [5]. This erythrocytic cycle is responsible for most of the pathological symptoms of malaria such as fever through the induction of a pro-inflammatory pyrogenic immune response [6].

Among the phagocytes, neutrophils are the first line of defense to respond to pathogens by generating reactive oxygen species, and antimicrobial peptides and proteases, or by neutrophil extracellular trap (NET) formation [7,8]. Studies have linked neutrophil activation and circulating NETs to the pathogenesis of malaria, including parasite sequestration in the microvasculature and endothelial dysfunction, resulting in impaired tissue perfusion and organ dysfunction [9,10,11]. A previous report demonstrated the presence of NETs in children with uncomplicated falciparum malaria with parasites trapped within NETs [12] and in malaria patients with severe disease [13,14]. However, there is a paucity of data on the direct interaction of HZ crystals and neutrophils, but the severity of malaria is associated with the clearance capacity of circulating HZ crystals by neutrophils [15]. HZ can interact with serum/plasma molecules such as proteins, lipids, and DNA, even before they encounter immune cells [16,17]. Whether human plasma can affect the HZ crystal clearing capacity by neutrophils is currently not known. We hypothesized that human plasma proteins would impair the ability of neutrophils to internalize HZ crystals.

## 2. Material and Methods

### 2.1. Isolation of Human Blood Neutrophils

Blood from human healthy individuals was collected in S-Monovette with lithium heparin (Sarstedt, Germany), and plasma was separated and neutrophils were isolated using standard dextran sedimentation followed by Ficoll–Hypaque density centrifugation procedures [18]. Neutrophils were suspended in Roswell Park Memorial Institute (RPMI) medium (0.5 × 10^5^ cells/200 µL or 2.5 × 10^5^ cells/mL) and seeded into 96-well or 24-well plates in a 5% carbon dioxide atmosphere at 37 °C for 30 min before stimulation. The study to obtain whole blood samples from healthy volunteers was approved by the local Ethical Review Board of the Medical Faculty at the Hospital of the Ludwig-Maximilians-University (LMU) Munich. Informed consent was obtained from all subjects.

### 2.2. Fluorescence Microscopy of Hemozoin Uptake

Human blood neutrophils were cultured ex vivo in the presence or absence of synthetic HZ (50 and 100 µg/mL, Invivogen, San Diego, CA, USA) in RPMI medium with or without 10%, 30%, or 50% human plasma in 8-well chamber slides (7.5 × 10^5^ cells/well, Nunc Lab-Tek, Sigma-Aldrich, Germany) for 1, 2, and 18 h. After incubation, cells were fixed using 4% paraformaldehyde for 10 min at room temperature, washed twice with Dulbecco’s Phosphate-Buffered Saline (D-PBS), and stained with phalloidin green for 40 min (165 nM, Sigma-Aldrich, indicates actin filaments). After membrane staining, cells were mounted with 4′,6-Diamidin-2-phenylindol (DAPI) (Sigma-Aldrich, indicates cell nuclei). The uptake of HZ by neutrophils was visualized using a Leica TL Light-emitting diodes (LED) fluorescence or confocal microscope (Leica, Wetzlar, Germany).

### 2.3. Uptake of Hemozoin in Neutrophils Using Flow Cytometry

Human blood neutrophils were cultured ex vivo in 96-well plates (0.5 × 10^5^ cells/200 μL) in the presence or absence of HZ (50 and 100 µg/mL) or silica crystals (200 μg/mL, Sigma-Aldrich, Germany) in RPMI medium without or with 10%, 30%, or 50% human plasma for 1, 2, and 18 h. In some experiments, cytochalasin D (10 µM, Sigma-Aldrich) was used to block phagocytosis of HZ crystals. To look at the effect of plasma proteins, HZ crystals were pre-incubated with or without fibrinogen (0.5 mg/mL, Sigma-Aldrich), albumin (3.25 mg/mL, Bethyl Labs, Montgomery, AL, USA), or Ringer’s solution (30%, negative control, Fresenius Kabi, Germany) for 30 min prior to stimulation with neutrophils. After stimulation, culture supernatants were collected and stored at −20 °C until further use, and cells harvested to quantify the percentage of cells that had internalized HZ crystals (HZ crystal^+^ neutrophils) were determined by flow cytometry using the BD FACSCalibur flow cytometer and FlowJo v7 software (Tree Star, Ashland, OR, USA).

To confirm intracellular uptake of HZ by neutrophils, human neutrophils were cultured with or without HZ (50 and 100 µg/mL) in RPMI medium for 2 h and then stained with the pHrodo red acetoxymethyl (AM) intracellular pH indicator (Thermo Fisher Scientific, Germany) for flow cytometry analysis as per the manufacturer’s protocol. An increase in the mean fluorescence intensity (MFI) of pHrodo red indicates an intracellular pH drop following HZ uptake.

### 2.4. Preparation and Stimulation of Neutrophils with Platelet-Poor Plasma

Human blood was drawn and standard dextran sedimentation performed to remove red blood cells. The top layer was removed and transferred into a 15 mL FALCON tube prior to centrifugation at 1500 *g* for 15 min. Using a new transfer pipette, the top layer was transferred into a new 15 mL FALCON tube and centrifuged at 1500 *g* for 15 min. After centrifugation, the top ¾ of plasma was removed using a transfer pipette and transferred into a new 15 mL FALCON tube. Isolated human blood neutrophils were then cultured in the presence or absence of synthetic HZ (50 and 100 µg/mL) in RPMI medium with or without 10%, 30%, or 50% human platelet-poor plasma for 2 h. After stimulation, neutrophils were either stained for fluorescence microscopy or harvested for flow cytometry to determine the percentage of HZ crystal^+^ neutrophils.

### 2.5. Morphological and Ultrastructural Analysis by Electron Microscopy

Blood neutrophils from healthy individuals were isolated and cultured (6.4 × 10^5^ cell/mL) ex vivo in the presence or absence of HZ (50 and 100 µg/mL) in RPMI medium with or without 30% human plasma for 2 h, and processed for scanning electron microscopy (SEM) and transmission electron microscopy (TEM).

For SEM, the samples were fixed in 2.5% glutaraldehyde in 0.1 M sodium cacodylate buffer, pH 7.2 at 4 °C for 24 h. Afterwards, the samples were adhered to poly-l-lysine-coated glass slides, dehydrated with increasing concentrations of ethanol (30% to 100%), followed by critical point drying in carbon dioxide to remove any water trace. Samples were then mounted on a stub and coated with gold. The analysis was carried out on a FEI Scios SEM (Hillsboro, OR, USA).

For TEM, the samples were fixed with 2.5% glutaraldehyde in 0.1 M sodium cacodylate buffer (pH 7.2) at 4 °C for 24 h. Post-fixation was performed using a solution of 1% osmium tetroxide, 0.8% potassium ferrocyanide, and 10.0 mM CaCl2 in 0.1 M cacodylate buffer for 1 h. Afterwards, samples were dehydrated in an increasing acetone gradient (30% to 100%) and embedded in Polybed 812 resin. Ultrathin sections were obtained, mounted on copper grids, and stained with uranyl acetate and lead citrate. Analysis was performed on a JEOL JM 1400 TEM (Jeol, Tokyo, Japan).

### 2.6. Statistical Analysis

Statistical analysis was performed using GraphPad Prism 7.0 software (GraphPad, San Diego, CA, USA). Data were compared either by one-way ANOVA with Tukey’s post-hoc test to calculate significance between three or more groups, or two-way ANOVA with Bonferroni’s comparison post-hoc test was carried out when using two parameters with multiple groups. Data are presented as mean ± SD. Differences were considered significant if *p* < 0.05. ns indicates not significant. Sample sizes are indicated in each corresponding figure legend.

## 3. Results

### 3.1. Neutrophils Took up Hemozoin Crystals under Serum/Plasma-Free Condition

HZ is thermally stable and insoluble, and can be visualized as a crystalline purple-black pigment, which is birefringent under polarized light (Figure S1A). Scanning electron microscopy (SEM) illustrated that HZ crystals had a size of 0.1–1 µm and were quite uniform in shape (Figure S1B) compared to other crystalline particles, including crystals of monosodium urate, calcium oxalate, and cholesterol, as well as asbestos fibers [19,20].

**Figure 1:**
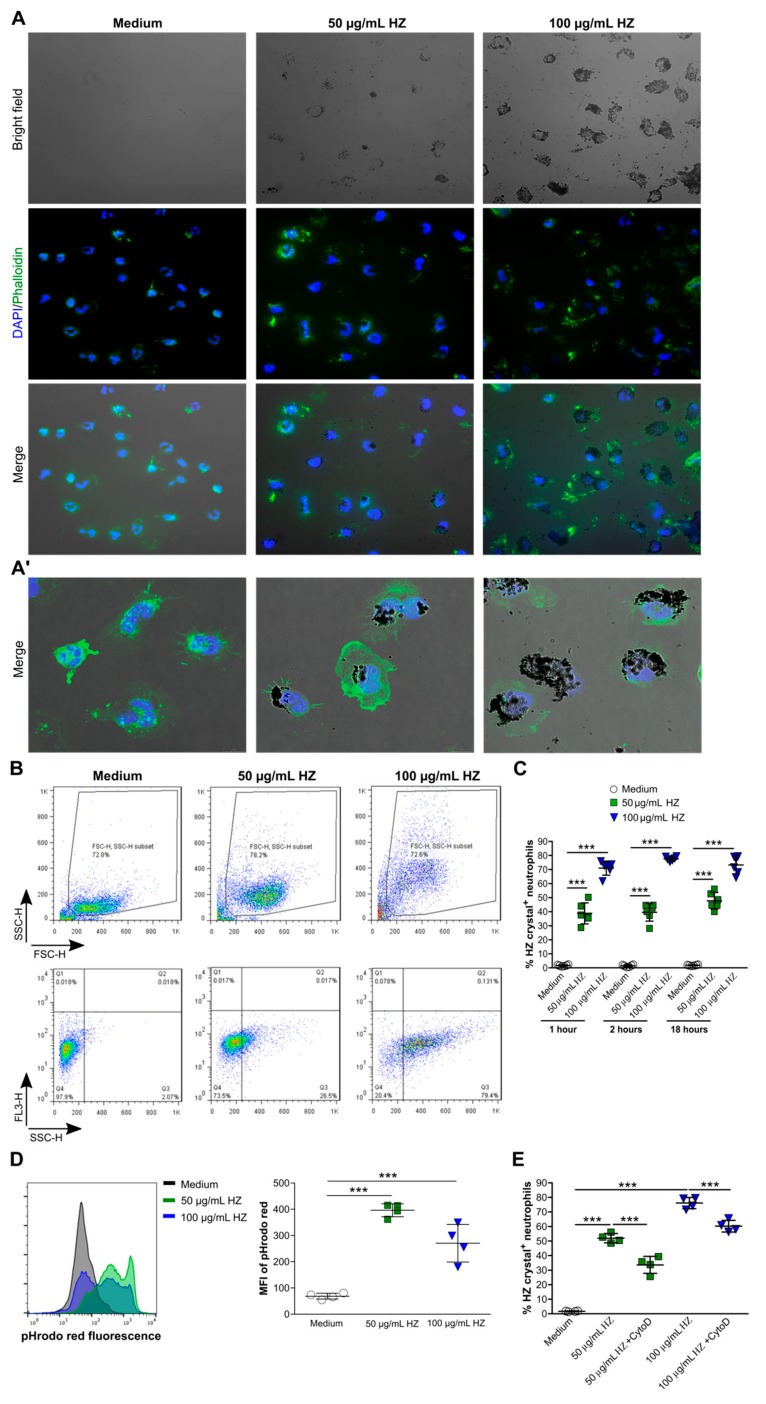
Neutrophils phagocytose large amounts of hemozoin (HZ) crystals. Human blood neutrophils were isolated from healthy volunteers and cultured in the presence or absence of hemozoin (HZ, 50 and 100 µg/mL) in Roswell Park Memorial Institute (RPMI) medium for up to 18 h, and fluorescence microscopy or flow cytometry were performed. (**A**,**A’**) Neutrophils were stained with phalloidin (green) and 4′,6-Diamidin-2-phenylindol (DAPI) (blue) for fluorescence microscopy (200× magnification (**A**)) and confocal microscopy (1000× magnification (**A’**)). (**B**) Representative images of the gating strategy to demonstrate the size of neutrophils (side scatter (SSC)-forward scatter (FSC)) and to quantify the percentage of neutrophils that had internalized HZ. (**C**) Percentage of HZ crystal^+^ neutrophils after 1, 2, and 18 h (*n* = 5–6 duplicates from 2–3 donors, two-way ANOVA). (**D**) Neutrophils were stained with the pH-sensitive dye pHrodo red and cultured with or without HZ. Quantification of HZ uptake in neutrophils was indicated by an increased mean fluorescence intensity (MFI) of pHrodo red using flow cytometry (*n* = 4 duplicates from 2 donors). (**E**) Percentage of HZ crystal^+^ neutrophils in the absence or presence of cytochalasin D (CytoD) (*n* = 4 duplicates from 2 donors, one-way ANOVA). Data are mean ± SD and representative of two independent experiments. *** *p* < 0.001; ns, not significant.

To investigate the capacity of neutrophils to internalize HZ, we cultured human blood neutrophils in the presence or absence of HZ crystals in RPMI medium for 2 h, and performed fluorescence microscopy using the actin stain phalloidin (green) and the nuclear marker DAPI (blue). As illustrated in Figure 1A,A’, the uptake of HZ resulted in morphological changes in neutrophils, in particular in size/shape, as indicated by phalloidin, and in deformed nuclei/granules, as indicated by DAPI. Flow cytometry analysis revealed that neutrophils increased in size and granularity following uptake of HZ, as shown by an increase in side scatter (SSC) (Figure 1B). The percentage of HZ crystal^+^ neutrophils increased in a dose-dependent manner but independent of the exposure time (Figure 1C).

Next, to confirm the internalization of HZ, human neutrophils were cultured in the presence or absence of HZ in RPMI medium, and the mean fluorescence intensity (MFI) of the pH-sensitive dye pHrodo red was determined via flow cytometry. As shown in Figure 1D, the uptake of HZ was associated with an increase in the MFI of pHrodo red in HZ-treated neutrophils compared to medium control after 2 h. Blocking HZ uptake with the inhibitor of actin polymerization cytochalasin D significantly reduced the percentage of HZ crystal^+^ neutrophils, although not completely, compared to untreated HZ crystal^+^ neutrophils (Figure 1E). Taken together, our data showed that neutrophils internalized HZ crystals partially via phagocytosis and partially via other uptake mechanisms.

### 3.2. Hemozoin Uptake Caused Nuclei Enlargement and Vesicle Formation but not NET Release

**Figure 2:**
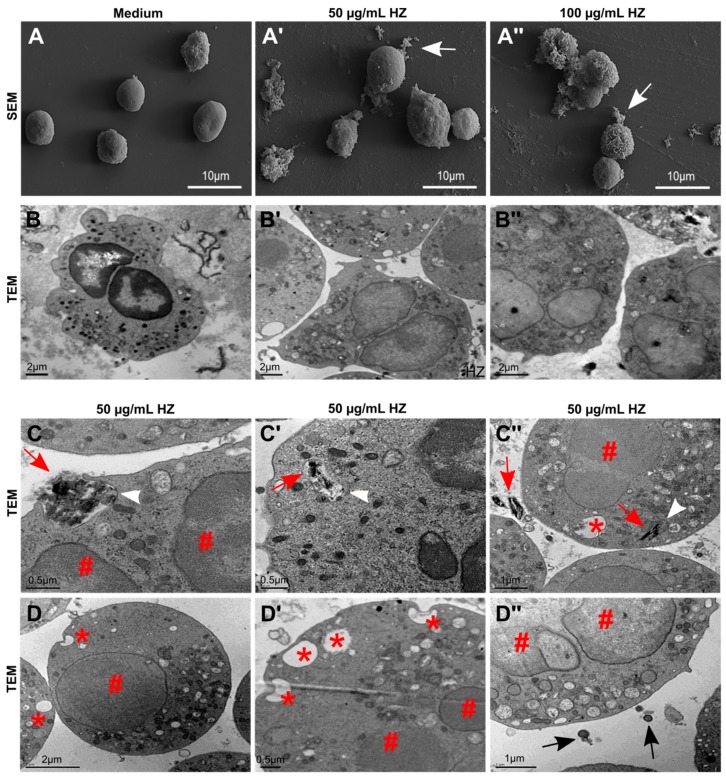
Hemozoin uptake induces nuclei enlargement, and intra- and extracellular vesicle formation in neutrophils but not neutrophil extracellular trap (NET) formation. Human neutrophils were isolated from healthy volunteers and cultured in the presence or absence of HZ (50 and 100 µg/mL) in RPMI medium for 2 h. (**A**–**C**) After stimulation, neutrophils were prepared for scanning and transmission electron microscopy (SEM and TEM, respectively). (**A**) SEM images of unstimulated neutrophils ((**A**) medium) and HZ-activated neutrophils, as indicated by a rough membrane surface and HZ clusters around neutrophils (white arrows) (**A**’,**A**”). (**B**) TEM images of unstimulated neutrophils (medium) showed dense nuclear hetero- and euchromatin, numerous granules, and few vesicles (**B**). Internalization of HZ led to loss of heterochromatin and nuclei enlargement (TEM, **B**’,**B**”). (**C**,**D**) TEM images of HZ-activated neutrophils showed large HZ aggregates (**C**, red arrows), HZ inside phagosomes (**C**’ and **C**”, indicated by white arrow heads), granules/endosomes (**C**”,**D**,**D**”), formation of vesicles/vacuoles (**C**”,**D**,**D**’, indicated by *), loss of heterochromatin (**C**,**C**”,**D**,**D**’,**D**”, indicated by #), breakdown of the nuclear membrane (**C**’), and release of extracellular vesicles (**D**”, indicated by black arrows).

To characterize the internalization process of HZ and the morphological abnormalities in more detail, we performed SEM and transmission electron microscopy (TEM) of human neutrophils in the presence or absence of HZ. Untreated neutrophils appeared round in shape with an even surface (Figure 2A, medium). After intracellular uptake of HZ, neutrophils increased in size and became activated, as illustrated by their rough membrane surface (Figure 2A’,A”). Neutrophils were also surrounded by clusters/aggregates of HZ crystals (white arrows) (Figure 2A’,A”). Under normal conditions (medium), neutrophils showed numerous dense granules and lysosomes in the cytoplasm (Figure 2B). The multi-lobed nucleus with the highly condensed heterochromatin (dark) was neatly marginalized to the edge of the nucleus, indicating intact cells (Figure 2B). However, internalization of HZ resulted in nuclei enlargement and loss of nucleus-associated heterochromatin, but the release of neutrophil extracellular traps (NETs) was not observed (Figure 2B’,B”). In addition, we observed that neutrophils ingested large HZ masses (Figure 2C, red arrows) into phagosomes (Figure 2C’,C”, as indicated by white arrow head). The uptake of HZ also resulted in nuclei enlargement with breakdown of the nuclear membrane (as indicated by #), the appearance of intracellular endosomes and lysosomes, and the release of extracellular vesicles (Figure 2D”, indicated by black arrows) as well as in the formation of vesicles/vacuoles to internalize smaller and single HZ crystals (Figure 2C”,D,D’, indicated by *). Thus, the intracellular uptake of HZ occurred via endocytosis into phagosomes and vesicles/vacuoles, which caused morphological changes in neutrophils without inducing cell membrane rupture or release of NETs.

### 3.3. Human Plasma Impaired Hemozoin Uptake in Neutrophils

**Figure 3:**
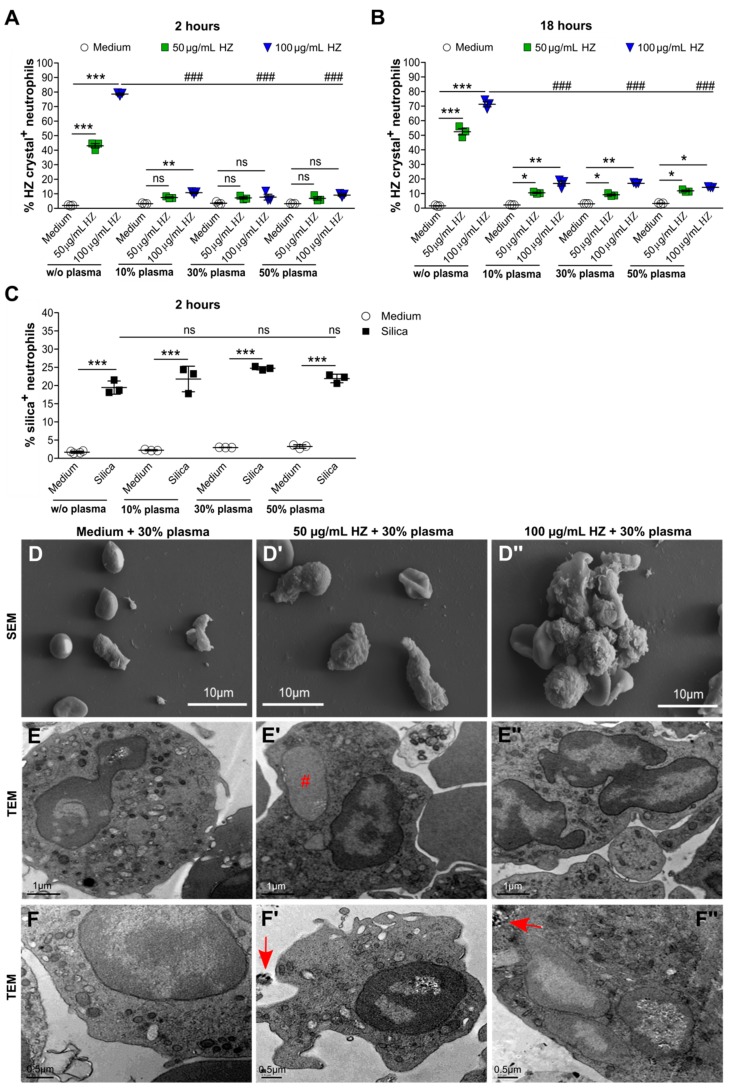
Human plasma impaired hemozoin but not silica uptake in neutrophils. Human neutrophils were isolated from healthy volunteers and cultured in the presence or absence of HZ (50 and 100 µg/mL) or with different amounts of human plasma for 2 and 18 h. (**A**,**B**) Percentage of HZ crystal^+^ neutrophils was determined using flow cytometry after 2 h (**A**) and 18 h (**B**) (*n* = 3 donors). (**C**) Neutrophils stimulated with or without silica crystals and/or human plasma for 2 h. The percentage of neutrophils that had taken up silica was determined by flow cytometry (*n* = 3 donors). Data are mean ± SD and representative of two independent experiments. * *p* < 0.05; ** *p* < 0.01; *** *p* < 0.001; ns, not significant using two-way ANOVA. w/o indicates without. (**D**) Scanning electron microscopy (SEM) images of neutrophils treated with 30% plasma and/or HZ of 2 h. (**E**,**F**) Transmission electron microscopy (TEM) images of neutrophils treated with 30% serum and/or HZ of 2 h. TEM images showed HZ-activated neutrophils with normal morphology and occasionally loss of nuclear heterochromatin (**E’**, indicated by #), and extracellular and intracellular HZ (**F’**,**F”**, indicated by red arrows). ### *p* < 0.001.

Recent studies reported that HZ crystals can interact with blood molecules such as proteins, lipids, and DNA in malaria [16,17]. However, the effect of protein-coated HZ on the ability of neutrophils to internalize these crystals is currently not known. Indeed, ex vivo cell culture experiments with medium might produce artificial results because the uptake of HZ in malaria occurs at a plasma concentration of around 50%. To investigate this, we cultured neutrophils with or without increasing amounts of human plasma from healthy individuals in the presence or absence of HZ for 2 and 18 h. Flow cytometric analysis revealed that neutrophils cultured without plasma (w/o plasma) took up large amounts of HZ after 2 h, whereas in the presence of human plasma (10–50%) the ability of neutrophils to recognize and internalize HZ was significantly inhibited (Figure 3A), even upon exposure up to 18 h (Figure 3B). In contrast, human plasma had no effect on the capacity of neutrophils to take up silica particles of the same size (Figure 3C). Morphologically, the presence of human plasma did not change the appearance of neutrophils (Figure 2A and Figure 3D–F). Those few neutrophils that had taken up HZ in the presence of plasma were activated, as indicated by their rough membrane surface (Figure 3D’,D”), and occasionally showed nuclei enlargement and loss of nuclear heterochromatin (Figure 3E’, indicated by #). Taken together, the data indicated that plasma altered the ability of neutrophils to recognize HZ as a danger signal, a vital mechanism during host defense.

### 3.4. Fibrinogen Altered the Uptake of Hemozoin by Human Neutrophils

**Figure 4:**
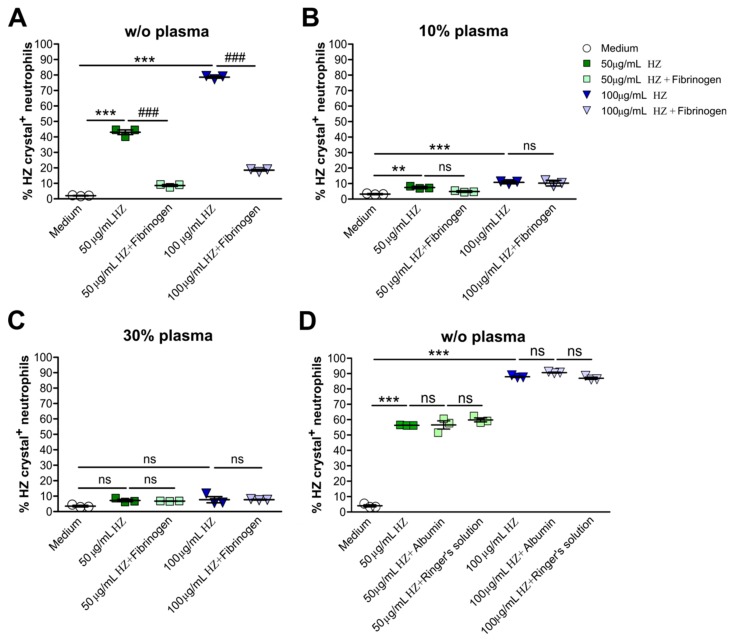
Fibrinogen impaired uptake of hemozoin by human neutrophils. Human neutrophils from healthy volunteers were cultured in the presence or absence of HZ (50 and 100 µg/mL) and/or with human plasma and/or fibrinogen for 2 h. (**A**–**C**) The percentage of HZ crystal^+^ neutrophils incubated in the absence or presence of fibrinogen without (w/o) plasma (**A**) or with 10% (**B**) and 30% (**B**) human plasma determined by flow cytometry (*n* = 3 donors). (**D**) Neutrophils were cultured in the presence or absence of HZ with or without albumin or Ringer’s solution in RPMI medium, and the percentage of HZ crystal^+^ neutrophils determined by flow cytometry after 2 h (*n* = 3 donors). Data are mean ± SD and representative of two independent experiments. ** *p* < 0.01; *** *p* < 0.001; ns, not significant using one-way ANOVA. ### *p* < 0.001.

In malaria, a variety of serum/plasma proteins bind to HZ crystals such as serum amyloid A, gelsolin, fibrinogen, albumin, and the lipopolysaccharide (LPS)-binding protein [16]. Among these, fibrinogen levels have also been shown to increase in children with malaria infection [21]. To test whether fibrinogen can influence the uptake of HZ in neutrophils, we pre-incubated HZ with or without fibrinogen prior to stimulation with neutrophils. Flow cytometric analysis revealed that fibrinogen significantly diminished the percentage of HZ crystal^+^ neutrophils compared to HZ-treated neutrophils only under the plasma-free condition (Figure 4A). However, in the presence of 10% and 30% human plasma, fibrinogen had no additional effect on the uptake of HZ by after 2 h of stimulation (Figure 4B,C, respectively). Unlike fibrinogen, albumin and Ringer’s solution (negative control) did not affect HZ uptake under the plasma-free condition (Figure 4D), suggesting that fibrinogen coating of HZ crystals inhibits the ability of neutrophils to internalize these crystals.

### 3.5. Removal of Platelets from Plasma Further Impaired HZ Uptake by Human Neutrophils

**Figure 5:**
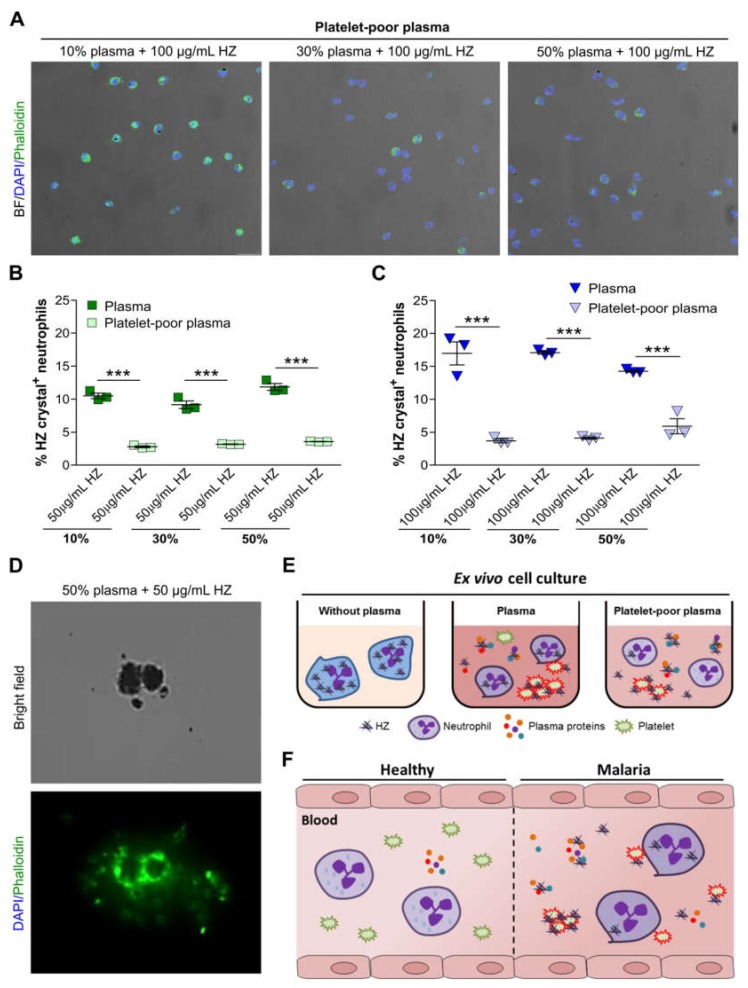
Platelet-poor plasma impaired hemozoin uptake by neutrophils. Human blood neutrophils were isolated from healthy volunteers and cultured with 10%, 30%, or 50% of platelet-poor plasma or plasma in the presence of HZ (50 and 100 µg/mL) for 2 h, and fluorescence microscopy or flow cytometry was performed. (**A**) Neutrophils cultured in platelet-poor plasma with HZ were stained with phalloidin (green) and DAPI (blue) for fluorescence microscopy. Images are shown as a merge of phalloidin and DAPI with bright field (BF) (200× magnification). (**B**,**C**) The percentage of neutrophils that had internalized HZ at concentrations of 50 µg/mL (**B**) and 100 µg/mL (**C**) in the presence of platelet-poor plasma or plasma were determined by flow cytometry (*n* = 3 donors). (**D**) Human plasma containing platelets was incubated with 50 µg/mL HZ and stained for phalloidin (green) for fluorescence microscopy (630× magnification). Data are mean ± SD and representative of two independent experiments. *** *p* < 0.001; ns, not significant using two-way ANOVA. (**E**) Schematic of ex vivo neutrophil culture experiments with plasma or platelet-poor plasma in the presence of HZ. (**F**) Schematic illustrating that during malaria HZ crystals bind to plasma proteins, which was associated with a diminished endocytotic capacity of neutrophils. Despite the impaired HZ uptake, those few neutrophils that did ingest HZ crystals relied on HZ-induced platelet activation.

To investigate whether platelets play a role during neutrophil HZ uptake, we cultured human neutrophils with plasma or platelet-poor plasma in the presence of HZ for 2 h. Fluorescence microscopy revealed that neutrophils internalized very few HZ crystals in human platelet-poor plasma (Figure 5A). This observation was in line with a significant decrease in the percentage of HZ crystal^+^ neutrophils in the presence of platelet-poor plasma compared with plasma only when cultured with 50 µg/mL HZ (Figure 5B) and 100 µg/mL HZ (Figure 5C). We also observed that the platelets in plasma accumulated around HZ and formed aggregates as illustrated by the positive actin staining for phalloidin and negative staining for the nuclear dye DAPI (Figure 5D). These data suggest that the uptake of HZ by neutrophils may depend on HZ-related platelet interaction.

## 4. Discussion

We hypothesized that human plasma would alter the ability of neutrophils to internalize malaria-related HZ crystals. Indeed, our ex vivo data revealed that the HZ internalization by neutrophils occurred via endocytosis into phagosomes as well as into vesicles/vacuoles (Figure 5E). Interestingly, plasma proteins including fibrinogen impaired this uptake process, whereas the presence of platelets enhanced it. These findings highlight the importance of factors regulating neutrophil endocytosis in vivo that are frequently ignored in ex vivo studies.

Neutrophils play an important role in the pathogenesis of malaria via processes including *Plasmodium* parasite killing and NET formation [11,12]. Recent reports have shown that heme, a known malaria danger-associated molecular pattern released during parasite egress, robustly induces NETs but not infected red blood cells, merozoites, and digestive vacuoles containing HZ, in tumor necrosis factor (TNF)-α-primed human neutrophils [14]. We found that human blood neutrophils internalized HZ in phagosomes and vesicles/vacuoles, which triggered morphological abnormalities without leading to cell membrane rupture and NET release. This suggests that although HZ does not directly induce NETs [14], they are known to significantly contribute to immune activation in other immune cells [5]. In contrast, very small nanoparticles (10 to 40 nm in size) and larger crystalline particles such as monosodium urate, calcium phosphate, cholesterol, and calcium oxalate crystals can induce mixed lineage kinase domain-like protein (MLKL)-driven neutrophil necroptosis and NET formation [19,22]. However, it is possible that neutrophils such as monocytes and macrophages remain viable after ingestion of HZ crystals, and that lysosome formation and acidification is normal [23], although HZ degradation might be impaired due to the inability of the lysosome to depolymerize HZ crystals. Thus, HZ can reside in these cells for long periods of time, but repeated phagocytosis or oxidative burst for further *Plasmodium* parasite killing is impaired [4,23,24,25], suggesting a state of sequestration of activated neutrophils [26]. Unresponsiveness of neutrophils in malaria accounts for increased susceptibility toward bacterial co-infections [27,28].

The process of endocytosis is characterized by polymerization of actin filaments and fusion of phagosomes with lysosomes to form phagolysosomes in macrophages [29] and human monocytes [30]. Unlike macrophages and monocytes, neutrophils do not form classical phagolysosomes and instead contain a large number of preformed granules that can rapidly fuse with phagosomes upon internalization of pathogens or larger amounts of particles [31]. Our data showed that human neutrophils ingested larger HZ crystal masses via direct uptake (phagocytosis) into phagolysosomes, whereas single and smaller HZ crystals might be internalized into vesicles/vacuoles via a different endocytotic uptake mechanism known as pinocytosis, due to the small size of HZ (0.1–1 µm). Previous studies have shown that pinocytosis does not require actin-dependent engulfment of small particles, for instance, zymosan, nanoparticles, or latex beads, by neutrophils [32], macrophages and endothelial cells [33], and non-phagocytic cells [34]. However, further studies are needed to confirm the clearance of HZ by neutrophils via pinocytosis.

The role of HZ-binding proteins in the recognition, immune modulation and physiological clearance of HZ in neutrophils remains to be elucidated. We report for the first time that human plasma from healthy individuals, specifically fibrinogen, impairs the ability of neutrophils to ingest HZ but not silica crystals ex vivo. This is in line with previous reports showing that blood proteins such as apolipoprotein E, serum amyloid A, LPS binding protein, complement factor H, albumin, and fibrinogen that were found to be elevated in malaria individuals are able to bind to HZ [16,17]. Hence, HZ-binding proteins alter the recognition of HZ as a danger signal for neutrophil clearance. The in vivo relevance of these findings remains to be proven, as the hematin-core crystal in HZ may remain shielded from serum proteins by the surrounding membranes/lipids [26,27].

It is known that circulating neutrophils and platelets interact during infection including malaria, inflammation, and thrombosis, and that they can modulate each other’s functions [35,36]. In malaria patients, circulating platelets and platelet-bound neutrophils are reduced, hence these complexes are either lost, the neutrophils migrate to tissues, or they form NETs [13]. Our ex vivo data showed that the ability of neutrophils to clear HZ crystals even further decreases in platelet-poor plasma compared to normal human plasma. This may imply that neutrophils require platelets for HZ clearance. Previous reports have shown that activated platelets can initiate or amplify various neutrophil responses including phagocytosis, production of oxygen radicals, and NET formation. Such responses are initiated either by a direct contact or by the release of soluble mediators such as chemokine (C-C motif) ligand 5 (CCL5) and platelet factor 4 [37,38]. In addition, platelet interactions enhance the phagocytic capacity of neutrophils towards various bacteria in vitro [39,40,41]. Conversely, neutrophils can also release soluble mediators such as cathepsin G and elastase that augment platelet responses by activation of protease-activated receptors on platelets [42,43,44]. However, further studies are needed to investigate the crosstalk between HZ-mediated platelet activation and HZ crystal uptake by neutrophil.

Limitations of our study are that we lack access to plasma from malaria-infected patients to investigate the impact of malaria-related plasma proteins on the phagocytic capacity of neutrophils to ingest HZ. As mentioned above, many plasma proteins that can bind to HZ have been identified in malaria patients [16], and it is possible that besides fibrinogen, other plasma proteins may alter the uptake of HZ by neutrophils. Furthermore, the role of HZ in modulation of host innate and inflammatory responses has been investigated using different HZ preparation protocols. HZ can be synthesized from hematin (sHZ) or natural HZ (nHZ), or digestive vacuoles containing hemozoin can be purified from infected red blood cells in culture [27]. We used synthetic HZ and not nHZ or digestive vacuoles of *Plasmodia* for our ex vivo cell culture experiments. Although sHZ and nHZ crystals are similar in size, and capable of inducing the same level of inflammation, sHZ with a smaller or larger crystal size may differently affect the function of neutrophils.

## 5. Conclusions

In conclusion, we found that the engulfment of HZ crystals by neutrophils via endocytosis relies on crystal–platelet interaction, whereas plasma proteins such as fibrinogen inhibit HZ crystal uptake and clearance from the extracellular space. HZ ingestion does not trigger the release of NETs, as reported for many other crystalline microparticles (Figure 5F). These data raise the question of how malaria-related thrombozytopenia may affect HZ-driven manifestations and outcomes during malaria infection.

## Supplementary Materials

The following are available online at https://www.mdpi.com/2073-4409/9/1/93/s1: Figure S1: Morphology of hemozoin crystals.
