# Supplementary material for: Plasma Proteins and Platelets Modulate Neutrophil Clearance of Malaria-Related Hemozoin Crystals
Source: Cells. 2019 Dec 30;9(1):93. doi: 10.3390/cells9010093 (PMC7017347; doi:10.3390/cells9010093)
Supplement: Supplementary file 1 [file cells-09-00093-s001.pdf]

Supplementary Materials

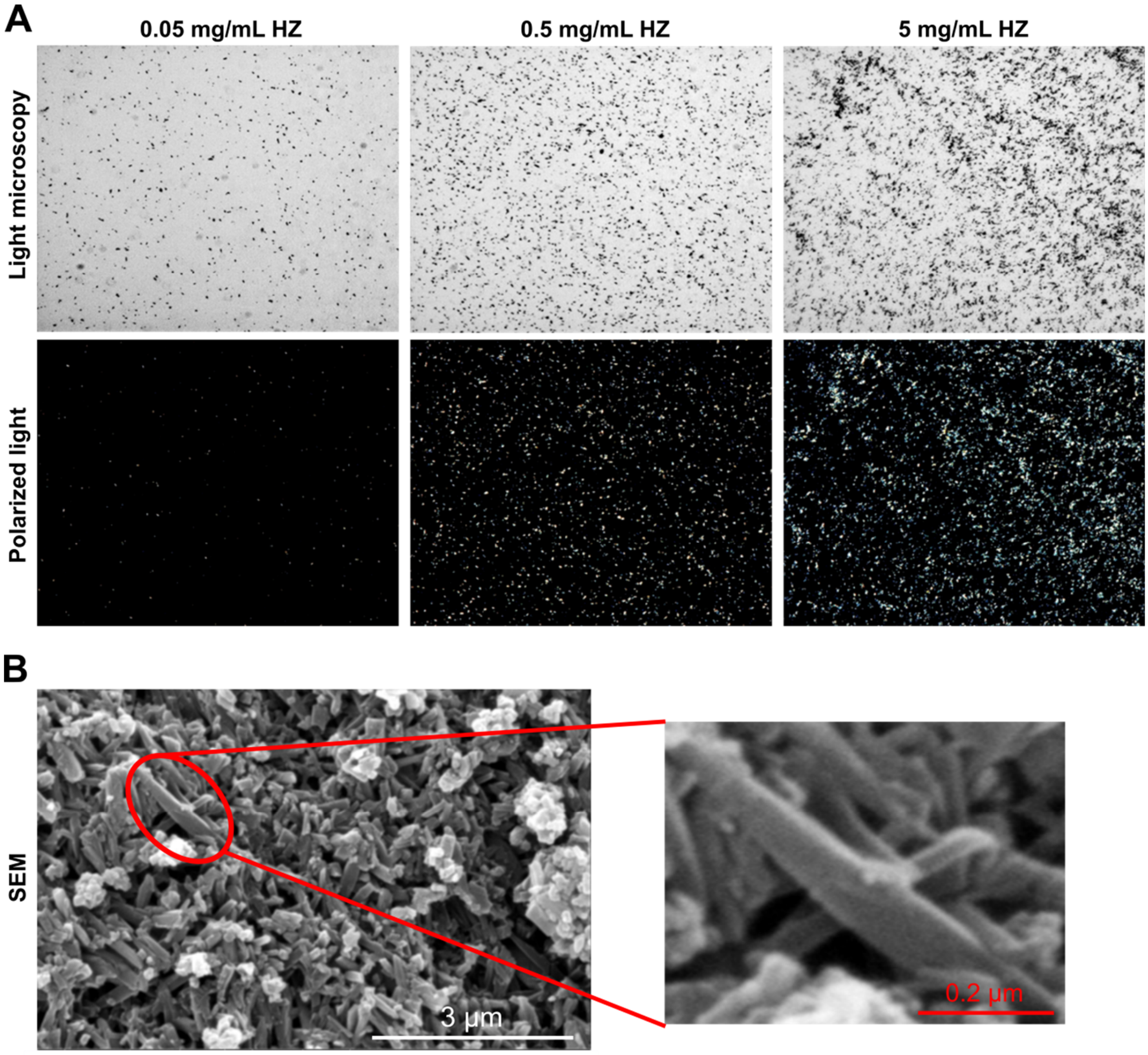

Figure S1: Morphology of hemozoin crystals. (**A**) Hemozoin (HZ) at different concentrations (0.05, 0.5 and 5 mg/mL) visualized as crystalline purple-black pigment under the light microscope, which is birefringent under polarized light (25× magnification). (**B**) Scanning electron microscopy (SEM) illustrates very small and uniform HZ crystals (0.1–1 µm in size).
